# A Comparative Analysis of Bi-Portal Endoscopic Spine Surgery and Unilateral Laminotomy for Bilateral Decompression in Multilevel Lumbar Stenosis Patients

**DOI:** 10.3390/jcm12031033

**Published:** 2023-01-29

**Authors:** Dong-Chan Eun, Yong-Ho Lee, Jin-Oh Park, Kyung-Soo Suk, Hak-Sun Kim, Seong-Hwan Moon, Si-Young Park, Byung-Ho Lee, Sang-Jun Park, Ji-Won Kwon, Sub-Ri Park

**Affiliations:** Department of Orthopedic Surgery, College of Medicine, Yonsei University, Seoul 120-752, Republic of Korea

**Keywords:** lumbar, MIS, BESS, ULBD, multilevel spinal stenosis

## Abstract

The clinical and radiological results before and after surgery were compared and analyzed for patients with multilevel lumbar stenosis who underwent bi-portal endoscopic spine surgery (BESS) and microscopic unilateral laminotomy for bilateral decompression (ULBD). We retrospectively identified 47 and 49 patients who underwent BESS and microscopic ULBD, respectively, who were diagnosed with multi-level lumbar stenosis. Clinical outcomes were evaluated using the visual analog scale score for both back and leg pain, and medication (pregabalin) use and Oswestry Disability Index (ODI) scores for overall treatment outcomes were used pre-operatively and at the final follow-up. Radiological outcomes were evaluated as the percentage of dura expansion volume, and percentage preservation of both facets and both lateral recess angles. The follow-up period of patients was about 17.04 months in the BESS group and about 16.90 months in the microscopic ULBD group. The back and leg visual analog scale (VAS) scores and average pregabalin use decreased more significantly in the BESS group than in the microscopic ULBD group (each *p*-value 0.0443, <0.001, 0.0378). All radiological outcomes were significantly higher in the BESS group than in the ULBD group. The change in ODI in two-level spinal stenosis showed a significantly higher value in the BESS group compared to the microscopic ULBD group (*p*-value 0.0335). Multilevel decompression with the BESS technique in multiple spinal stenosis is an adequate technique as it shows better clinical and radiological results than microscopic ULBD during a short-term follow-up period.

## 1. Introduction

Degenerative lumbar disease is accompanied by back pain, radiating leg pain, or intermittent claudication, which greatly affects the health and quality of life of patients. Surgical treatment is considered in patients with long-term symptoms who do not respond to conservative treatment, and typically involves simple decompression, decompression, or fusion. For patients with spondylolisthesis or foraminal stenosis, spinal fusion is required; however, decompression-only surgery is sufficient for patients with neurological disorders due to central stenosis or lateral recess stenosis. Decompression usually involves symptom-causing laminotomy and ligamentum flavectomy. In classical decompression, the spinous process and interspinous ligament are removed to reveal the lamina; however, this may cause back pain or segmental instability [1,2,3,4].

The treatment of multilevel lumbar spinal stenosis is controversial. Because neurological symptoms do not occur in all radiological stenotic lesions, there is always controversy about appropriate treatment in patients with multilevel spinal stenosis. As a result, some surgeons choose extensive laminectomy, although it may cause segmental instability. Therefore, other surgeons perform multilevel decompression and fusion to avoid iatrogenic spinal instability, and ensure long-term efficacy; however, adjacent segment disease (ASD) is common after posterior lumbar spinal fusion. Therefore, it is necessary to decompress on appropriate stenotic lesions based on the patient’s symptoms [5,6,7,8].

Several minimally-invasive surgery (MIS) alternatives have been proposed to overcome these problems, such as bi-portal endoscopic spine surgery (BESS) and microscopic unilateral laminotomy for bilateral decompression (ULBD) [1,2]. In particular, BESS is known to be a safe and effective surgical method because of its small skin incision, low tissue damage in the surgical area, and spinal canal decompression in the enlarged surgical field. Several studies have reported that single-level MIS decompression of lumbar stenosis leads to improved post-operative functional outcomes and improved clinical scores [1,2]. However, clinical data and functional outcomes after MIS for multilevel lumbar stenosis are lacking. Also, until now, there have only been a few cases where multilevel decompression was performed using an endoscope. This study aimed to retrospectively compare and analyze the clinical and radiological results before and after surgery in patients with two or more segments of lumbar spinal stenosis who underwent BESS decompression and microscopic ULBD; as well, we hypothesized that BESS decompression provides better results with multilevel lumbar spinal stenosis.

## 2. Materials and Methods

### 2.1. Patient Selection

After approval by the institutional review board (approval number 2022-0459-001), 47 patients who underwent spinal canal decompression using BESS and 48 patients who underwent spinal canal decompression using microscopic ULBD, and who were diagnosed with more than two levels of lumbar spinal stenosis between March 2019 and December 2021, were retrospectively identified. In order to include patients who were followed up for at least 1 year, patients who underwent surgery until the 2nd week of December 2021 were included. The follow-up period of patients was 17.04 ± 2.41 months (mean ± SD) in the BESS group and 16.90 ± 2.43 months in the microscopic ULBD group. The study was performed in a single center; BESS was performed by a single surgeon, and microscopic ULBD was performed by two surgeons for 24 cases, for a total of 48 microscopic ULBD cases. The clinical symptoms before surgery included low back pain, intermittent neurogenic claudication, radiating pain, numbness, and weakness in the lower extremities. All patients underwent simple lumbar radiography, computed tomography (CT), and magnetic resonance imaging (MRI) to diagnose lumbar spinal stenosis, and the target patients underwent conservative treatments such as medication, physical therapy, and selective nerve block for more than 6 weeks; however, neurological symptoms did not improve. If multilevel spinal stenosis was shown on MRI, selective nerve blocks were performed at each level to find correlation with patient’s symptoms and determine the level of decompression. Exclusion criteria were as follows: (1) Patients with segmental instability; (2) degenerative scoliosis above 20° Cobb’s angle; (3) severe foraminal stenosis; (4) spondylolisthesis of grade 2 or higher; (5) infection, tumors; (6) follow-up of less than 12 months; (7) previous lumbar surgery were excluded.

### 2.2. Surgical Procedure

#### 2.2.1. BESS

Surgery was performed with the patients in the prone position under general or epidural anesthesia. Before draping, the interlaminar and disc spaces of the surgical site, pedicle, and spinous process through the C-arm were marked to create appropriate working and viewing portal locations. A skin incision was made approximately 1 cm inside the pedicle medial border; the portals could be made higher or lower in the interlaminar space because the disc level and interlaminar space level may differ. Depending on the degree of lordosis or degenerative changes, it was necessary to adjust the incision slightly. Subsequently, using a muscle detacher, the soft tissues of the interlaminar space were sufficiently detached to create a working space and determine whether fluid outflow occurred.

Hemostasis was performed using radiofrequency (RF) or bone wax to control epidural vein or cancellous bone bleeding to ensure good visibility. Using a 4-mm high speed diamond burr, we first drilled the ipsilateral lamina and spinous process base portion to create more space to allow for the movement of the instruments. An ipsilateral laminotomy was performed cranially until the origin of the ligamentum flavum (LF), the “ligamentum flavum notch,” was reached. We then drilled the upper margin of the lower lamina for easier detachment of the LF. We checked the ipsilateral lateral margin of the LF with a dura dissector or blunt root hook while drilling the hypertrophic facet joint medial margin to prevent the ipsilateral facet joint. Using a blunt hook, the LF was cut in half from the LF notch to the caudal portion of the epidural fat layer and the ipsilateral LF was removed. The LF was separated from the undersurface of the contralateral lamina using a dura dissector or a tiny RF. When the LF was detached, there was sufficient space to insert the burr between the lamina and LF. The undersurface of the contralateral lamina was drilled until the lateral recess was reached. After removal of the contralateral LF, the lateral margin of the dural sac and traversing root pathway were checked. If the space in that area was inadequate, further removal of the LF attached to the SAP, the bony ingrowth portion in the SAP base, or the bony structure around the pedicle, was performed to make space using straight or curved upward Kerrison punches. The osteophytic ridge of the disc could be decompressed using a shoe-shaped bone impactor and curved upward Kerrison punch, or a rotatory Kerrison punch was used to remove a slightly more hypertrophic osteophyte under the surface of the lower lamina.

After the dural sac was sufficiently expanded, the lateral margin of the dural sac was confirmed to be sufficient, and the area in which the traversing root entered the pedicle was expanded appropriately. With a 0° endoscope, it was possible to check the exit root and the SAP tip. The hypertrophied SAP tip was removed using Kerrison punches or a hockey chisel osteotome to identify the exiting root with greater certainty after removal of the foraminal ligament.

**Figure 1:**
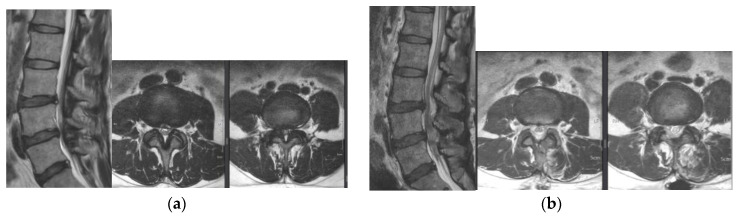
Pre-operative (**a**) and post-operative (**b**) MR images of a 76-year-old female patient with L3–4–5 spinal stenosis who underwent BESS surgery.

In the case of intervertebral foraminal disc herniation, this was also removed. By changing the 0° endoscope to a 30° endoscope, we could improve our view of the medial area of the ipsilateral facet joint. Using the 30° endoscope, we exposed more lateral margin space of the ipsilateral dural sac and traversing root, and checked the ipsilateral SAP tip and exiting root pathway. Using the hockey chisel osteotome, a curette, and Kerrison punches, we performed the same procedure on the contralateral side to create sufficient space to traverse the root pathway, dural sac lateral area, and exiting root pathway area. The same procedure was performed at each level (Figure 1).

#### 2.2.2. Microscopic ULBD

With the patient prone on the spinal table, we used an image intensifier to determine the incision position and then position the retractor of choice to identify the inferior aspect of the superior lamina. Surgeons used ×2.5 or ×3.5 loupe magnification or the operative microscope. Beginning with the laminotomy on the approach side, we drilled to identify the LF, and removed bone up to the superior attachment of the LF. To gain access to the contralateral side of the canal for bilateral decompression, enough spinous process was removed to gain access to the midline and contralateral LF. The superior aspect of the decompression usually corresponded to superior LF attachment; removal of the upper limit of the LF provided an important landmark to confirm the superior limit of decompression.

**Figure 2:**
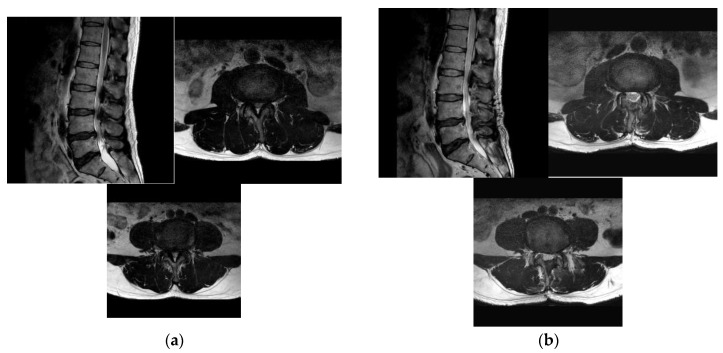
Pre-operative (**a**) and post-operative (**b**) MR images of a 72-year-old female patient with L3–4–5 spinal stenosis who underwent microscopic ULBD surgery.

Detachment of the LF from the facet joint on the approach side was performed using a combination of angled curettes and Kerrison rongeurs; a partial medial facetectomy or removal of adequate facet hypertrophy on the approach side was necessary to expose the traversing nerve root. Decompression of the thecal sac on the contralateral side of the canal is a potentially dangerous aspect of the procedure, with the highest risk of dural injury and cerebrospinal fluid leakage; it is used to create enough room on the ipsilateral side so that instruments can be safely introduced into the canal for contralateral decompression. Reducing paraspinal muscle dissection substantially reduces iatrogenic muscle injury and blood loss, and oozing from bone removal can be easily controlled with bone wax or a variety of hemostatic agents (Figure 2).

### 2.3. Outcome Evaluation

Clinical outcomes were evaluated using the visual analog scale (VAS) score for both back and leg pain and medication (pregabalin) use. Although there are various types of medication used for spinal stenosis, we investigated the amount of pregabalin used in relation to radiculopathy symptoms to evaluate the improvement of radiculopathy after surgery. These values were assessed pre-operatively and post-operatively at 1, 3, 6, and 12 months. Additionally, the Oswestry Disability Index (ODI) score for overall treatment outcomes was calculated pre-operatively and at the final follow-up. Radiological outcomes were evaluated as the percentage of dura expansion volume, percentage preservation of both facets, and both lateral recess angles using pre-operative and post-operative MRI performed to evaluate the results of decompression within 3 days following surgery.

The dural expansion volume was measured using an imaginary line encircling the area between the facet joint and lamina on the axial image at the most stenotic level to evaluate the decompression result. The lateral recess angle was defined as the angle between the floor of the lateral recess and the LF on the ventral side of the inferior articular process. The percentage of facet preservation was defined as a comparison between the length of the post-operative facet and the length of the pre-operative facet.

### 2.4. Statistical Analysis

For the patient demographic data, continuous variables are expressed as representative values as mean and standard deviation, and an independent two-sample *t*-test was used to test the difference in the group. Categorical variables are representative values expressed as N and percentages, and the chi-square test or Fisher’s exact test was used to test the differences between groups. The independent two-sample-test method was used to determine whether there was a difference in hospital day (HOD), operation (OP) time, and OP bleeding between groups.

To determine whether there was a difference between the percentage of dura expansion volume, percentage preservation of both facets, and both lateral recess angles between groups, the independent two-sample t-test method was used. For each level (2 level & 3 level), a subgroup analysis was also performed. To evaluate the difference between the groups, within the group in the back VAS, leg VAS, and the amount of medication measured by time point, we analyzed the association between the data measured at various points from the same participant. There was a slight difference in pre-op values between the two groups, and it was adjusted and compared. If the outcome was a continuous variable, the linear mixed model was applied for analysis; if it was a dichotomous type, generalized estimating equations were used. When the pre-op ODI value was corrected, ANCOVA was performed using the pre-op value as a correction variable, post-op ODI as an outcome, and group as an independent variable to compare the ODI after surgery between the BESS group and the microscopic ULBD group.

All statistical analyses were performed using SAS version 9.4 (SAS Institute, Inc. Cary, NC, USA). Differences in continuous and non-continuous variables between the groups were analyzed using independent two-sample t-tests and chi-square tests. The linear mixed model through repeated-measures two-way analysis of variance was performed to determine whether the measured clinical outcomes differed based on the evaluation periods between or within groups. Statistical significance was set at *p* < 0.05.

## 3. Results

### 3.1. Patient Demographics

The patient characteristics and descriptive statistics of the patient groups are summarized in Table 1. A total of 59 females and 39 males were included, with a median age of 68 years.

For the range of surgical levels, 84 patients had two levels and 11 patients had three levels operated upon. The numbers of spondylolisthesis and scoliosis patients were 29 (30.53%) and 8 (8.42%), respectively. Comparison of patient information between the two groups showed no statistical differences in all variables.

Post-operative complications occurred in three patients in the BESS group and five patients in the microscopic ULBD group. In the BESS group, a dural tear appeared in one patient and epidural hematoma appeared in two patients. In the microscopic ULBD group, dural tears appeared in three patients and epidural hematoma appeared in two patients; however, there were no re-operations or sequelae in all patients. We were able to confirm epidural hematoma well enough because post-operative MRIs were taken within three days. Neither of the hematoma cases in both groups were large in size. Therefore, patients did not complain of more pain than the previous radiation pain or temporarily complained of radiation pain to other areas; however, all of them gradually decreased. Also, no neurological deficits were observed, and additional management was not required.

### 3.2. Surgical Outcome Evaluation

The patient surgical outcomes and descriptive statistics between the patient groups by surgical level range are summarized in Table 2. In the total level group, the average hospitalization duration was approximately 4.4 days for patients who underwent BESS and 6.9 days for the microscopic ULBD group. The average operation time was approximately 112 min in patients who underwent BESS and 91 min in patients who underwent microscopic ULBD. The average intraoperative blood loss was approximately 93 cc in the BESS group and 143 cc in the microscopic ULBD group. Hospitalization period, operation time, and blood loss showed statistically significant differences. When examining the differences according to the number of levels, the two levels showed the same statistical results as the total level. However, in the group of three levels, the hospitalization period showed the same statistical results as those for total levels and two levels; however, there was no statistically significant difference in the operation time and blood loss.

### 3.3. Radiologic Outcome Evaluation

The pre- and post-operative MRI results were compared as percentages. In the BESS group, the average dural sac diameter increased by 198.2%, the ipsilateral side of the facet joint was approximately 91.2%, and the contralateral side of the facet joint was preserved by 93.4% at the total level. The ipsilateral side lateral recess angle increased by approximately 151.7% and the contralateral side increased by 159.2% at the total level. In the microscopic ULBD group, the average dural sac diameter increased by 181.5%; the ipsilateral side of the facet joint was 80.9%; and the contralateral side of the facet joint was preserved by 84.538% at the total level. The ipsilateral side lateral recess angle increased by approximately 131.5% and the contralateral side increased by 126.6% at the total level. There was a statistically-significant difference between the two groups in the expansion ratio of the dural sac, ipsilateral side facet joint preservation ratio, and increase in the contralateral side lateral recess angle. Similar statistical results were observed for levels 2 and 3 (Table 3).

### 3.4. Clinical Outcome Evaluation

**Figure 3:**
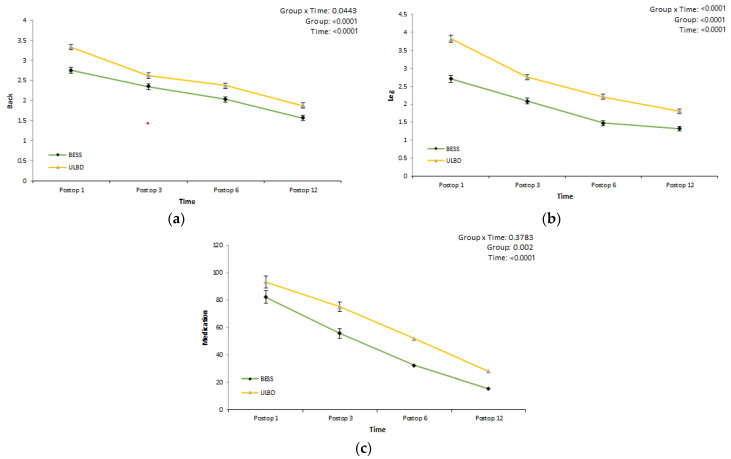
Clinical outcomes: VAS score and medication use by range of levels. Back (**a**); leg (**b**); medication (mg) (**c**).

Using a linear mixed model, we determined whether there was a difference in back and leg VAS scores between the two groups after surgery and whether there was a difference in drug use (Table 4, Figure 3). The back VAS scores in the BESS and microscopic ULBD groups decreased to 1.558 and 1.871, respectively, at 12 months after surgery. There was a statistically-significant difference in the improvement of lower back pain between the two groups over time. The leg VAS scores in the BESS and microscopic ULBD groups decreased to 1.323 and 1.809, respectively, at 12 months after surgery. There was a statistically-significant difference in leg pain improvement between the two groups over time. Average pregabalin drug use decreased to 15.282 mg and 27.745 mg in the BESS and microscopic ULBD groups, respectively, at 12 months after surgery. The amount of drug used also showed a statistically-significant difference between the two groups over time.

Level of function (disability) in activities of daily living was evaluated before and 12 months after surgery using the ODI. The ODI scores in the BESS and microscopic ULBD groups decreased to 18.258 and 19.851, respectively, at final follow-up at the total level. There was a statistically significant difference in two levels decompression; however, there were no statistically significant differences based on the range of the surgical levels (Table 5).

## 4. Discussion

Until now, multilevel spinal stenosis has been mainly diagnosed by radiological methods such as MRI and, based on this, surgical treatment has been determined. Adilay et al. set the criteria for multilevel spinal stenosis as follows: MRI or CT confirmation of compressive multilevel spinal stenosis as central sagittal diameter < 12 mm), with or without lateral recess stenosis (lateral recess < 3 mm). Also, they considered multilevel decompressive laminectomy if difference in the canal diameter between the most stenotic level and the second stenotic level was <3 mm, even though the second stenotic level diameter was >9 mm [5]. Schizas et al. described a classification from A to D based on the morphology of the dural sac, as observed on T2 axial magnetic resonance images based on the rootlet/cerebrospinal fluid ratio [9]. Using this grading system, Soman et al. reported that morphological grading is a useful tool in deciding whether to perform surgery for multilevel spinal stenosis. Grade C and D stenosis should be decompressed, whereas grades A and B should not be, unless clinically justified [10]. Yoshikane et al. defined Schizas’ grade B as grade M (moderate) and grade C and D as grade S (severe). They reported that multilevel grade S stenosis can be a risk factor for re-operation in patients who underwent selective single level lumbar endoscopic unilateral laminotomy for bilateral decompression (LE-ULBD) [6].

In our study, multilevel spinal stenosis basically classified grades from A to D using MRI axial images in the same way as for the above-mentioned grades. In the case of grades C and D, surgical treatment was attempted; however, most importantly, surgical treatment was performed not only for radiological finding but also for levels consistent with the patient’s symptoms through other tests.

However, radiological findings are not necessarily consistent with symptoms. Amundsen et al. found no relationship between the degree of stenosis (measured on myelography and CT) and clinical symptoms in patients selected from a neurology department on the basis of clinical symptoms of spinal stenosis [11]. Also, Sirvanci et al. examined the correlation between imaging and ODI in 63 surgical candidates with spinal stenosis. They studied cross-sectional areas and subjective criteria of lateral recess and foraminal stenosis on axial MRI scans and found no significant correlation between those parameters and ODI [12].

Therefore, it is very important to determine the levels where surgical treatment should be performed in multilevel spinal stenosis. Consequently, in our study, the standard of multilevel decompression was not judged only by radiological findings. We, first, accurately identified the patient’s symptoms through physical examination and compared them with levels of severe, then moderate, degree on MRI axial images; also, selective nerve block was performed to determine whether the levels matched the patient’s symptoms or improved symptoms. Furthermore, we performed EMG /NCV and varicose vein ultrasound for differential diagnosis of other diseases. For this reason, unlike other studies, we believed that clinical improvement would have been excellent when performing multilevel decompression.

For multilevel lumbar spinal stenosis, the choice of the surgical levels remains controversial. Also, there are only a few publications that have undergone multilevel decompression in multilevel spinal stenosis. Takahashi et al. suggested that multilevel laminectomy delays the resolution of cauda equina adhesions, which may lead to the onset of certain clinical symptoms [13]. Moreover, radiological stenosis does not always cause symptoms, as it can present, to some extent, in asymptomatic patients [14]. Adilay et al. reported that a poor pre-operative ODI score in the multilevel decompression group and a poor response to surgery compared with the single level decompression patients may have simply been due to a higher burden of degenerative change and all of the multiple complaints and symptoms that can accompany such change [5]. Murata et al. performed selective micro-endoscopic laminectomy (MEL), addressing only symptomatic levels in multilevel stenosis with residual remaining lumbar stenosis in radiographic findings. They reported that selective MEL could have similar effects without increased re-operation rates; also, surgeons may consider more limited selective decompression in patients with multilevel stenosis [15]. Ulrich et al. reported multi-segmental stenotic cases in which a single-level decompression was associated with a significantly more favorable spinal stenosis measurement of symptom and function scores, as compared with multilevel decompression [16].

On the other hand, Lee et al. performed endoscopic decompression in lumbar spinal stenosis in five studies and a meta-analysis of 156 patients, including 26 patients who underwent multilevel decompression. All of the patients showed good clinical results, and only 1.9% of patients underwent revision surgery [17]. Khanna et al. performed multilevel decompression with the MIS technique using a microscope or loupe in 92 patients with multiple stenosis and reported that the results were clinically effective and durable. Fusion rates remained low within two years, being 8.6%, compared with the literature data for open procedures [7].

In multilevel spinal stenosis, symptoms remain continuously after surgical treatment or spondylolisthesis occurs, resulting in re-operation, which has been reported in several studies. Adilay et al. found that 4 of 64 patients who underwent multilevel decompressive laminectomy and all patients underwent instrumented fusion because of instability [5]. Lee et al. reported that the re-operation rate after LE-ULBD was 1.9% in their meta-analysis, in which selective single-level surgery was performed for single and multilevel spinal stenosis in a total of 156 patients from five studies [17]. Yoshikane et al. reported that re-operation was performed in 10.2% of the patients with multilevel spinal stenosis who underwent selective single level decompression [6]. Because damage to the poster supporting structure caused by the open decompression surge is unavoidable, wide removal of facet joints also results in potential complications such as re-operation for segmental instability, a long recovery time, and rehabilitation [18,19,20].

Various attempts have been made to solve this problem [16,21,22], and a method called ULBD, using several MIS tools, has been used. Oertel et al. reported long-term results of ULBD with a mean follow-up duration of 5.6 years. One hundred and thirty patients (97.7%) improved immediately after surgery, 94 (92.2%) of the 102 patients available for long-term follow-up examination remained improved, and the incidence of complications was 9.8% [23]. There is significant evidence that MIS techniques applied to single-level lumbar pathology result in decreased estimated blood loss, tissue injury, and fewer perioperative complications [24]. In these aspects, the use of MIS techniques is appealing when developing surgical treatment plans for such patients. However, the questions surrounding MIS applicability to multilevel stenosis involve its ability to effectively decompress the canal and whether that improvement is durable without the development of structural complications sooner than with open procedures [7]. Khanna et al. reported that their study population who received multilevel MIS decompression also demonstrated sustained and statistically-significant improvement beyond the minimum clinically importance difference (MCID) in patient-reported outcome measures (PROM) categories at the three-month, six-month, one-year, and two-year time points [7].

More surgical techniques and instruments have been developed, and endoscopic spine surgery can be a good alternative to open microscopic decompression, as this endoscopic technology is free from the aforementioned potential results. Lim et al. reported that the clinical outcome of percutaneous stenoscopic lumbar decompression (PSLD) appeared to be better compared with that of MED and open laminectomies, and shorter hospitalization and lower complication rates were observed when compared to PSLD and MEL [8].

BESS has been rapidly evolving in recent years. Compared to the instruments used for microscopic or full-endoscopic surgery, bi-portal endoscopic instruments may be less costly, while the learning curve is comparable [25]. All the MIS approaches aim to obtain adequate decompression while preserving the integrity of the facet joint complex. Facet undercutting has been suggested to avoid excessive facet joint destruction. However, such techniques were difficult for open, tubular retractor-assisted, or micro-endoscopic approaches, because the surgeon’s visual point remained outside of the patient’s body or outside of the lamina. However, with the BESS technique, the surgeon’s visual point can be advanced inside of the lamina or into the contralateral lateral recess and the contralateral foramen, meaning that the surgeon can check for the offending pathological structures without visual limitation [26].

Our study was very meaningful because it is the first study comparing BESS techniques with existing MIS techniques for multilevel decompression in multilevel spinal stenosis.

This study showed a longer operation time and less hospital days, as well as less blood loss in the BESS group than in microscopic ULBD group. Compared to the results of other studies, it was found that the operation time was longer when endoscopic surgacy was performed. The reason for this is that, in order to make more area for the lateral recess area in our study, we spend enough time decompression on the hypertrophic bony structures, ligament flavum and disc materials of the path where the traversing roots passes. Also, in hospital days, most of BESS group patients started walking the day after surgery, and microscopic ULBD group patients also started walking on the second day after surgery. However, we allowed discharge when there were no specific findings on the lab and wound dressing was done about one or two times.

In radiologic outcomes, including post-operative dural sac expansion, ipsilateral and contralateral decompression were superior in the BESS group compared to the microscopic ULBD group. This difference may be attributed to being able to see the narrow three-dimensional view of the lateral recess using a 0° and 30° scope, as well as a magnified endoscopic view. We think this method can more easily and precisely decompress and preserve posterior structures and facet joint complex than other open or MIS techniques.

Clinical outcomes showed that both operations show favorable results in post-operative consecutive follow-up. The VAS scores of the back and leg were significantly lower in the BESS group than in the microscopic ULBD group. In addition, the observed medication doses were lower in the BESS group than in the microscopic ULBD group. This seems to be due to the maximized undercutting of both facet joints while maintaining the paraspinal and bony structures as much as possible in the case of the endoscope.

There are several non-surgical treatments for patients with spinal stenosis. Among them, drug treatments are often taken by mixing NSAIDs, oral corticosteroids, muscle relaxants, prostaglandin E1 analogs, anti-depressants, and anti-convulsants (gabapentin). Eguchi et al. reported that the combined use of neurotropin and limaprost showed an additional effect on walking speed compared with single drug administration; as well, neurotropin might contribute to the improvement of low back pain, walking speed, and standing balance [27].

In our study, opioid drugs were not used before and after surgery, and only nonsteroidal anti-inflammatory drugs (NSAIDs) and limaprost with gabapentin were used for patients with back pain and radiculopathy symptoms. Symptoms remaining after surgery were controlled only with pregabalin and NSAIDs. We think that it was a meaningful result to confirm the effect of pregabalin in patients with radiculopathy before and after decompression surgery.

The reason why ODI, Op time, blood loss, and HOD results do not show statistically-significant differences at the three levels in this study is probably due to insufficient patients numbers. We expect that these data will become significantly meaningful if more patients are added in the next long-term follow-up study.

This study has several limitations. First, since one orthopedic surgeon performed BESS, surgeon technical bias may be included; therefore, research involving other surgeons for the surgery is will be needed. Also, there was a difference between the surgeon who performed BESS and the surgeons who performed microscopic ULBD, which is an additional limitation of this study. However, there was no difference in areas other than surgery, because pre-operative treatment or post-operative management proceeded in the same process, according to the hospital protocol.

Second, the retrospective study design limits further evaluation of the detailed patient information. In this study, we focused on comparing and analyzing the differences in clinical and radiological outcomes of multilevel decompression using the BESS technique and mircoscopic ULBD technique; therefore, we did not collect all of the patients’ details such as BMI, smoking history, and underlying diseases. In the future long-term follow-up study, it is planned to make the comparison target between groups more accurate using propensity matching.

Third, the follow-up date was short to investigate post-operative long-term complications and re-operation rates. Actually, in our study, the follow-up period of patients was 17.04 ± 2.41 months (mean ± SD) in the BESS group and 16.90 ± 2.43 months in the microscopic ULBD group. In this period, we could not find instability or symptoms deteriorating in both groups. Of course, this value is also a short period of time and the biggest limitation of our study; more than two-year long term follow-up data should be documented to verify our study. However, we expect that patients who have undergone multilevel decompression through the BESS technique, which sufficiently preserves facet joints, minimizes posterior structure damage, and can perform sufficient depression, can maintain their current status well without instability or the recurrence of symptoms.

## 5. Conclusions

The BESS technique with multilevel decompression in patients with multilevel spinal stenosis is expected to be an adequate technique, as it shows better clinical and radiological results than microscopic ULBD during a short-term follow-up period.

## Figures and Tables

**Table 1:** Patient demographics.

	Total (N = 95)	BESS (N = 47)	ULBD (N = 48)	*p*-Value
	Mean ± SD or N (%)	Mean ± SD or N (%)	Mean ± SD or N (%)	
Age	68.716 ± 8.736	68.234 ± 11.040	69.188 ± 5.734	0.6002
Gender				0.1222
F	56 (58.95)	24 (51.06)	32 (66.67)	
M	39 (41.05)	23 (48.94)	16 (33.33)	
Range of surgical levels				0.7768
2	84 (88.42)	42 (89.36)	42 (87.50)	
3	11 (11.58)	5 (10.64)	6 (12.50)	
Spondylolisthesis				0.2372
No	66 (69.47)	30 (63.83)	36 (75.00)	
Yes	29 (30.53)	17 (36.17)	12 (25.00)	
Scoliosis				0.1586
No	87 (91.58)	41 (87.23)	46 (95.83)	
Yes	8 (8.42)	6 (12.77)	2 (4.17)	
Post-operative complication				
No	87 (91.58)	44 (93.62)	43 (89.58)	
Yes	8 (8.42)	3 (6.38)	5 (10.42)	

F, female; M, male; SD, standard deviation.

**Table 2:** Surgical outcome by range of surgical levels.

**Total Level**				
	**Total (N = 95)**	**BESS (N = 47)**	**ULBD (N = 48)**	***p*-Value**
	**Mean** **±** **SD or N (%)**	**Mean** **±** **SD or N (%)**	**Mean** **±** **SD or N (%)**	
Hospital day	5.695 ± 1.963	4.426 ± 0.903	6.938 ± 1.929	<0.0001
Operation time (min)	101.032 ± 22.401	111.660 ± 14.073	90.625 ± 24.212	<0.0001
Operation bleeding (cc)	117.789 ± 56.567	92.553 ± 20.105	142.500 ± 68.874	<0.0001
**Two Levels**				
	**Total (N = 84)**	**BESS (N = 42)**	**ULBD (N = 42)**	***p*-Value**
	**Mean** **±** **SD or N (%)**	**Mean** **±** **SD or N (%)**	**Mean** **±** **SD or N (%)**	
Hospital day	5.571 ± 1.947	4.357 ± 0.850	6.786 ± 1.982	<0.0001
Operation time (min)	99.667 ± 21.949	111.619 ± 14.675	87.714 ± 21.616	<0.0001
Operation bleeding (cc)	112.619 ± 55.599	88.333 ± 15.526	136.905 ± 69.343	<0.0001
**Three Levels**				
	**Total (N = 11)**	**BESS (N = 5)**	**ULBD (N = 6)**	***p*-Value**
	**Mean** **±** **SD or N (%)**	**Mean** **±** **SD or N (%)**	**Mean** **±** **SD or N (%)**	
Hospital day	6.636 ± 1.912	5.000 ± 1.225	8.000 ± 1.095	0.0020
Operation time (min)	111.455 ± 24.147	112.000 ± 8.367	111.000 ± 33.311	0.9459
Operation bleeding (cc)	157.273 ± 49.818	128.000 ± 20.494	181.667 ± 55.287	0.0716

BESS, bi-portal endoscopic spine surgery; ULBD, unilateral laminomy for bilateral de-compression; SD, standard deviation.

**Table 3:** Radiological outcomes by range of surgical levels.

**Total**				
	**Total (N = 205)**	**BESS (N = 99)**	**ULBD (N = 106)**	***p*** **-Value**
	**Mean ± SD or N (%)**	**Mean ± SD or N (%)**	**Mean ± SD or N (%)**	
Expansion ratio of dural sac (%)	189.546 ± 56.683	198.152 ± 64.297	181.509 ± 47.421	0.0374
Ipsilateral Facet joint Preservation ratio (%)	85.878 ± 7.097	91.192 ± 3.079	80.915 ± 6.126	<0.0001
Contralateral facet joint Preservation ratio (%)	88.805 ± 7.076	93.374 ± 2.427	84.538 ± 7.329	<0.0001
Ipsilateral lateral recess angle increasing ratio (%)	141.298 ± 22.254	151.737 ± 21.104	131.547 ± 18.648	<0.0001
Contralateral lateral recess angle Increasing ratio (%)	142.366 ± 26.793	159.202 ± 19.954	126.642 ± 22.500	<0.0001
**Two levels**				
	**Total (N = 172)**	**BESS (N = 84)**	**ULBD (N = 88)**	***p*** **-Value**
	**Mean ± SD or N (%)**	**Mean ± SD or N (%)**	**Mean ± SD or N (%)**	
Expansion ratio of dural sac (%)	192.430 ± 59.008	204.881 ± 66.491	180.545 ± 48.306	0.0070
Ipsilateral Facet joint Preservation ratio (%)	85.657 ± 7.135	91.024 ± 3.208	80.534 ± 5.990	<0.0001
Contralateral facet joint Preservation ratio (%)	88.843 ± 7.118	93.405 ± 2.425	84.489 ± 7.397	<0.0001
Ipsilateral lateral recess angle Increasing ratio (%)	142.599 ± 22.565	151.583 ± 22.501	134.023 ± 19.117	<0.0001
Contralateral lateral recess angle Increasing ratio (%)	142.436 ± 26.297	159.226 ± 20.009	126.409 ± 21.102	<0.0001
**Three levels**				
	**Total (N = 33)**	**BESS (N = 15)**	**ULBD (N = 18)**	***p*** **-value**
	**Mean ± SD or N (%)**	**Mean ± SD or N (%)**	**Mean ± SD or N (%)**	
Expansion ratio of dural sac (%)	174.515 ± 39.978	160.467 ± 30.577	186.222 ± 43.809	0.0443
Ipsilateral Facet joint Preservation ratio (%)	87.030 ± 6.890	92.133 ± 2.066	82.778 ± 6.612	<0.0001
Contralateral facet joint Preservation ratio (%)	88.606 ± 6.955	93.200 ± 2.513	84.778 ± 7.191	0.0001
Ipsilateral lateral recess angle Increasing ratio (%)	134.515 ± 19.487	152.600 ± 10.736	119.444 ± 9.532	<0.0001
Contralateral lateral recess angle Increasing ratio (%)	142.000 ± 29.678	159.067 ± 20.335	127.778 ± 29.091	0.0014

**Table 4:** Clinical outcomes—VAS score and medication use by range of levels.

**Back**				
**Time**	**Group = BESS (0)** **Estimated Mean (SE)**	**Group = ULBD (1)** **Estimated Mean (SE)**	**Effect**	**Overall** ***p*-Value**
Postop 1 month	2.749(0.070)	3.329(0.069)	Group	<0.0001
Postop 3 month	2.345(0.072)	2.621(0.071)	Time	<0.0001
Postop 6 month	2.026(0.065)	2.371(0.064)	Group*time	0.0443
Postop 12 month	1.558(0.067)	1.871(0.067)		
**Leg**				
**Time**	**Group = BESS (0)** **Estimated Mean (SE)**	**Group = ULBD (1)** **Estimated Mean (SE)**	**Effect**	**Overall** ***p*-Value**
Postop 1 month	2.706(0.098)	3.809(0.097)	Group	<0.0001
Postop 3 month	2.089(0.077)	2.746(0.076)	Time	<0.0001
Postop 6 month	1.472(0.075)	2.205(0.074)	Group*time	<0.0001
Postop 12 month	1.323(0.063)	1.809(0.062)		
**Medication (mg)**				
**Time**	**Group = BESS (0)** **Estimated Mean (SE)**	**Group = ULBD (1)** **Estimated Mean (SE)**	**Effect**	**Overall** ***p*-Value**
Postop 1 month	82.303(3.089)	93.370(3.057)	Group	0.002
Postop 3 month	55.707(5.795)	75.141(5.734)	Time	<0.0001
Postop 6 month	32.303(4.468)	51.703(4.422)	Group*time	0.0378
Postop 12 month	15.282(3.425)	27.745(3.389)		

**Table 5:** Clinical outcomes: ODI change by range of levels.

**Total**				
	**BESS (N = 47)**	**ULBD (N = 48)**	**Difference (SE)**	***p*** **-Value**
	**Estimated Mean (SE)**	**Estimated Mean (SE)**		
Post-Op ODI	18.258(0.581)	19.851(0.575)	−1.593(0.819)	0.0548
**Two levels**				
	**BESS (N = 47)**	**ULBD (N = 48)**	**Difference (SE)**	***p*** **-Value**
	**Estimated Mean (SE)**	**Estimated Mean (SE)**		
Post-Op ODI	18.099(0.0.612)	19.973(0.612)	−1.874(0.867)	0.0335
**Three levels**				
	**BESS (N = 47)**	**ULBD (N = 48)**	**Difference (SE)**	***p*** **-Value**
	**Estimated Mean (SE)**	**Estimated Mean (SE)**		
Post-Op ODI	18.998(2.030)	19.502(1.830)	−0.503(2.918)	0.8673

## Data Availability

Not applicable.
